# Supplementary material for: Epithelial GPR35 protects from Citrobacter rodentium infection by preserving goblet cells and mucosal barrier integrity
Source: Mucosal Immunol. 2022 Mar 9;15(3):443–58. doi: 10.1038/s41385-022-00494-y (PMC9038528; doi:10.1038/s41385-022-00494-y)
Supplement: Supplementary file 1 — Supplementary information [file 41385_2022_494_MOESM1_ESM.pdf]

Figure S1

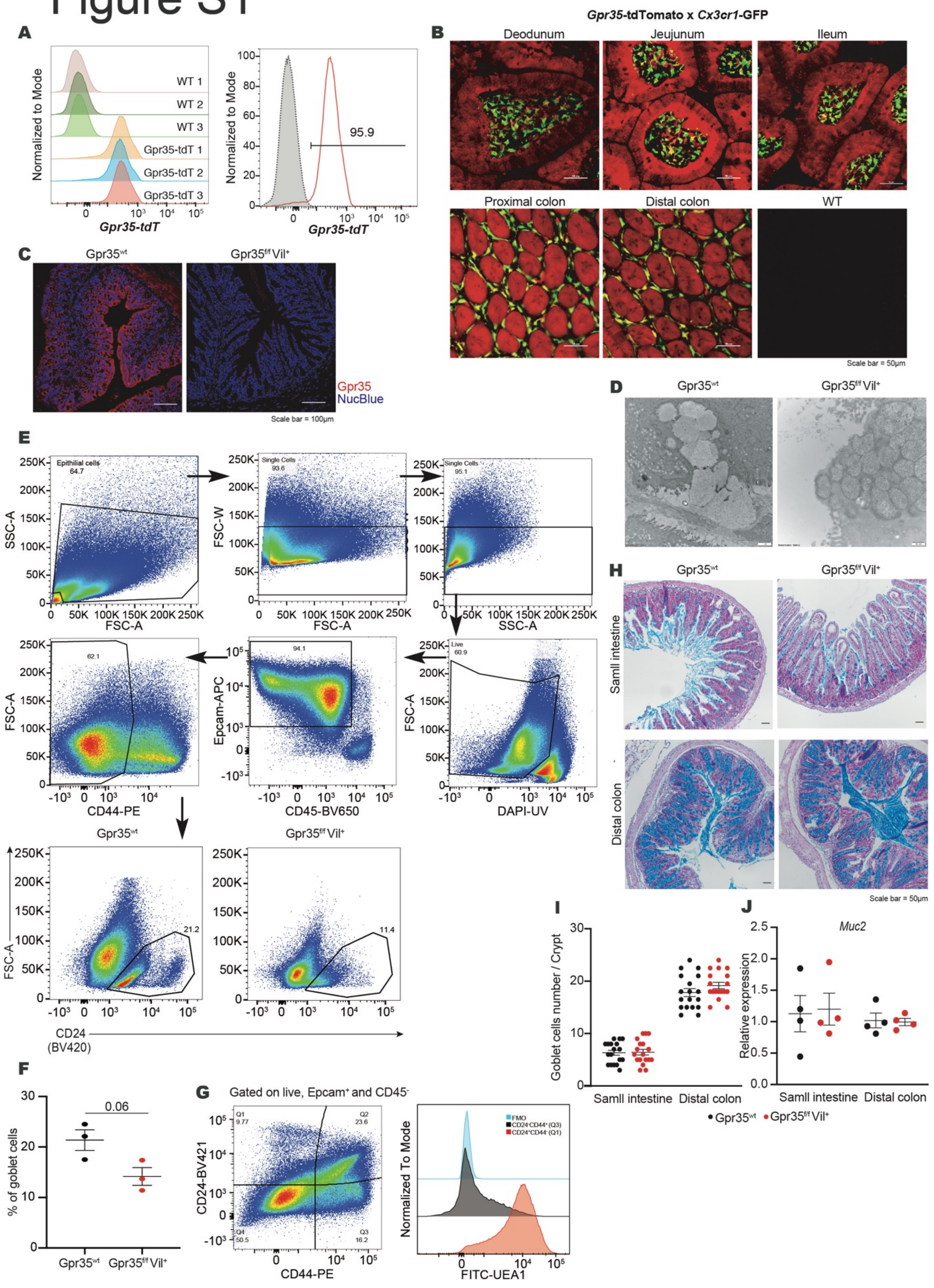

A
Normalized to Mode
WT 1
WT 2
WT 3
Gpr35-tdT 1
Gpr35-tdT 2
Gpr35-tdT 3
Gpr35-tdT
95.9
B
Gpr35-tdTomato x Cx3cr1-GFP
Deodunum
Jeujunum
Ileum
Proximal colon
Distal colon
WT
Scale bar = 50μm
C
Gpr35wt
Gpr35f/f Vil+
Gpr35
NucBlue
Scale bar = 100μm
D
Gpr35wt
Gpr35f/f Vil+
E
Epithilial cells
64.7
Single Cells
93.6
Single Cells
95.1
SSC-A
FSC-A
FSC-W
Live
60.9
94.1
62.1
Epcam-APC
CD45-BV650
DAPI-UV
CD44-PE
Gpr35wt
Gpr35f/f Vil+
21.2
11.4
CD24
(BV420)
H
Gpr35wt
Gpr35f/f Vil+
Samll intestine
Distal colon
Scale bar = 50μm
I
Goblet cells number / Crypt
Samll intestine
Distal colon
J
Muc2
Relative expression
Samll intestine
Distal colon
Gpr35wt
Gpr35f/f Vil+
F
% of goblet cells
0.06
Gpr35wt
Gpr35f/f Vil+
G
Gated on live, Epcam+ and CD45-
CD24-BV421
CD44-PE
Q1 9.77
Q2 23.6
Q3 16.2
Q4 50.5
Normalized To Mode
FMO
CD24-CD44+ (Q3)
CD24+CD44- (Q1)
FITC-UEA1

Figure S1. Epithelial cell-specific *Gpr35* deletion reduces goblet cell numbers

(A) Representative *Gpr35*-tdTomato expression by flow cytometry in epithelial cells ($Epcam^+$, $CD45^-$) from the proximal colon of *Gpr35*-tdTomato reporter mice (red unfilled histograms) and $Gpr35^{wt}$ mice (gray histograms) as the background control.

(B) *Ex vivo* fluorescence imaging of duodenum, jejunum, ileum, proximal colon and distal colon from *Gpr35*-tdTomato (red) x *Cx3cr1*-GFP (green) double reporter mice. The last panel shows the colon from a WT mouse as the background control.

(C) Confocal immunofluorescence images of proximal colon sections obtained from $Gpr35^{f/f}Vil^+$ and $Gpr35^{wt}$ littermates and stained for Gpr35 (red) and DAPI (blue). Scale bars, 100 μm.

(D) Transmission electron microscopy analysis of goblet cell morphology in the proximal colon of $Gpr35^{f/f}Vil^+$ and $Gpr35^{wt}$ co-housed littermates. Scale bars, 5 μm.

(E) Flow cytometry analysis of goblet cell percentages in the proximal colon of $Gpr35^{f/f}Vil^+$ and $Gpr35^{wt}$ co-housed littermates.

(F) Quantification of data from (E) indicating the percentages of goblet cells (n = 3) in each group.

(G) Flow cytometry analysis of UEA1 percentage in the $CD24^-CD44^+$ and $CD24^-CD44^+$ populations.

(H) Representative AB/PAS staining of small intestine and distal colon sections obtained from $Gpr35^{f/f}Vil^+$ and $Gpr35^{wt}$ co-housed littermates. Scale bars, 50 μm.

(I) Cell count of goblet cells in (H) performed blindly by two different investigators in at least 30 crypts.

(J) mRNA expression levels of *Muc2* measured by RT-qPCR in small intestine and distal colon samples obtained from $Gpr35^{f/f}Vil^+$ (n = 4) and $Gpr35^{wt}$ co-housed littermates (n = 4).

Data are represented as mean ± SEM ns, not significant by Mann-Whitney test.

Figure S2

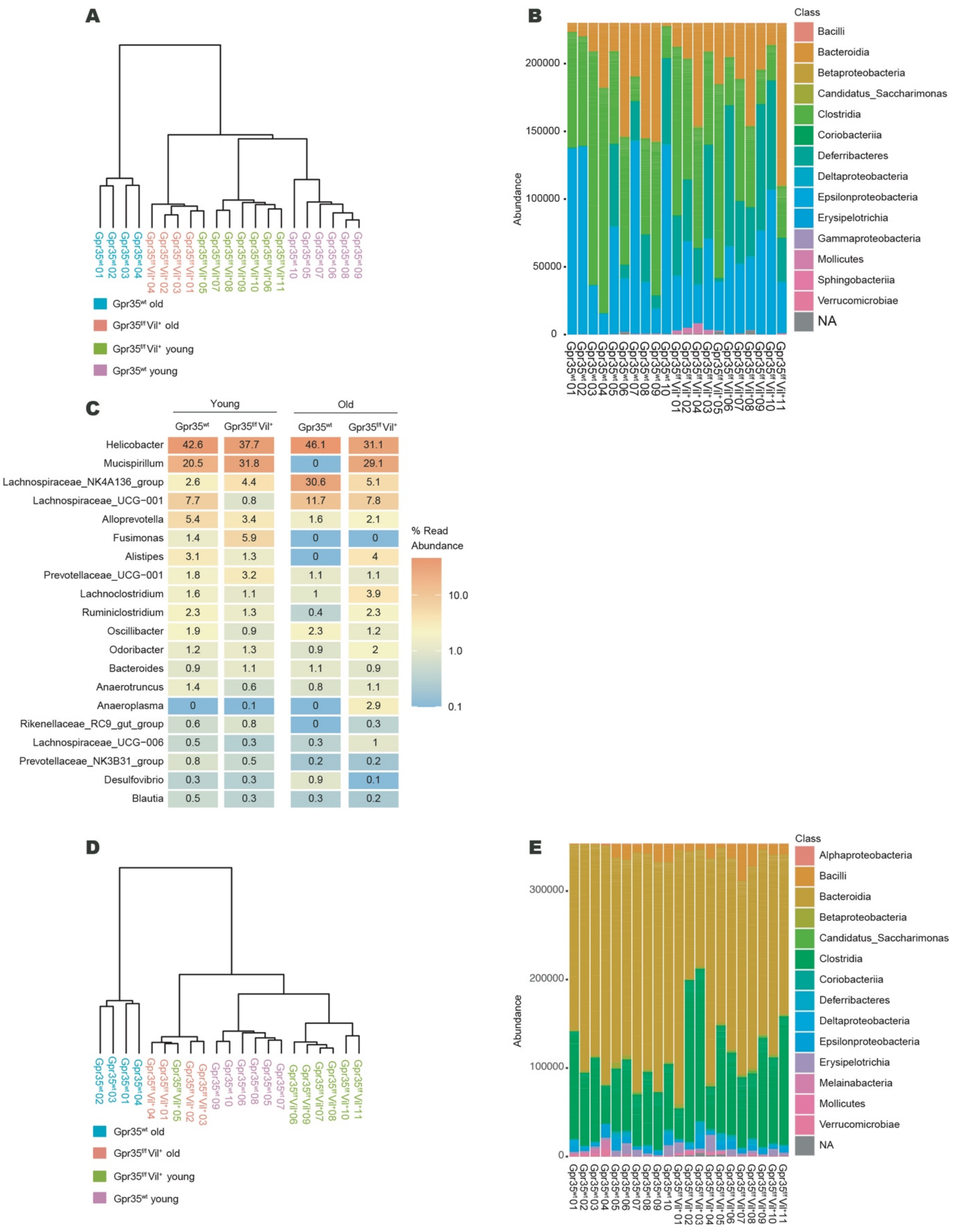

	Young		Old	
	Gpr35wt	Gpr35f/f Vil+	Gpr35wt	Gpr35f/f Vil+
Helicobacter	42.6	37.7	46.1	31.1
Mucispirillum	20.5	31.8	0	29.1
Lachnospiraceae_NK4A136_group	2.6	4.4	30.6	5.1
Lachnospiraceae_UCG−001	7.7	0.8	11.7	7.8
Alloprevotella	5.4	3.4	1.6	2.1
Fusimonas	1.4	5.9	0	0
Alistipes	3.1	1.3	0	4
Prevotellaceae_UCG−001	1.8	3.2	1.1	1.1
Lachnoclostridium	1.6	1.1	1	3.9
Ruminiclostridium	2.3	1.3	0.4	2.3
Oscillibacter	1.9	0.9	2.3	1.2
Odoribacter	1.2	1.3	0.9	2
Bacteroides	0.9	1.1	1.1	0.9
Anaerotruncus	1.4	0.6	0.8	1.1
Anaeroplasma	0	0.1	0	2.9
Rikenellaceae_RC9_gut_group	0.6	0.8	0	0.3
Lachnospiraceae_UCG−006	0.5	0.3	0.3	1
Prevotellaceae_NK3B31_group	0.8	0.5	0.2	0.2
Desulfovibrio	0.3	0.3	0.9	0.1
Blautia	0.5	0.3	0.3	0.2

Figure S2. Epithelial cell-specific *Gpr35* deletion correlates with an altered mucosa-associated microbiome

(A) Hierarchical clustering of mucosal bacterial samples based on Bray-Curtis dissimilarity and Ward's minimum variance method (ward.D2).

(B) Relative abundance of mucosal bacterial communities at the class level in $Gpr35^{f/f}Vil^{+}$ and $Gpr35^{wt}$ mice at different age points.

(C) Relative abundance of taxonomic groups at the genus level averaged across mucosa-associated bacteria samples of old and young $Gpr35^{f/f}Vil^{+}$ and $Gpr35^{wt}$ littermates.

(D) Hierarchical clustering of fecal bacterial samples based on Bray-Curtis dissimilarity and Ward's minimum variance method (ward.D2).

(E) Relative abundance of fecal bacterial communities at the class level in $Gpr35^{f/f}Vil^{+}$ and $Gpr35^{wt}$ mice at different age points.

Figure S3

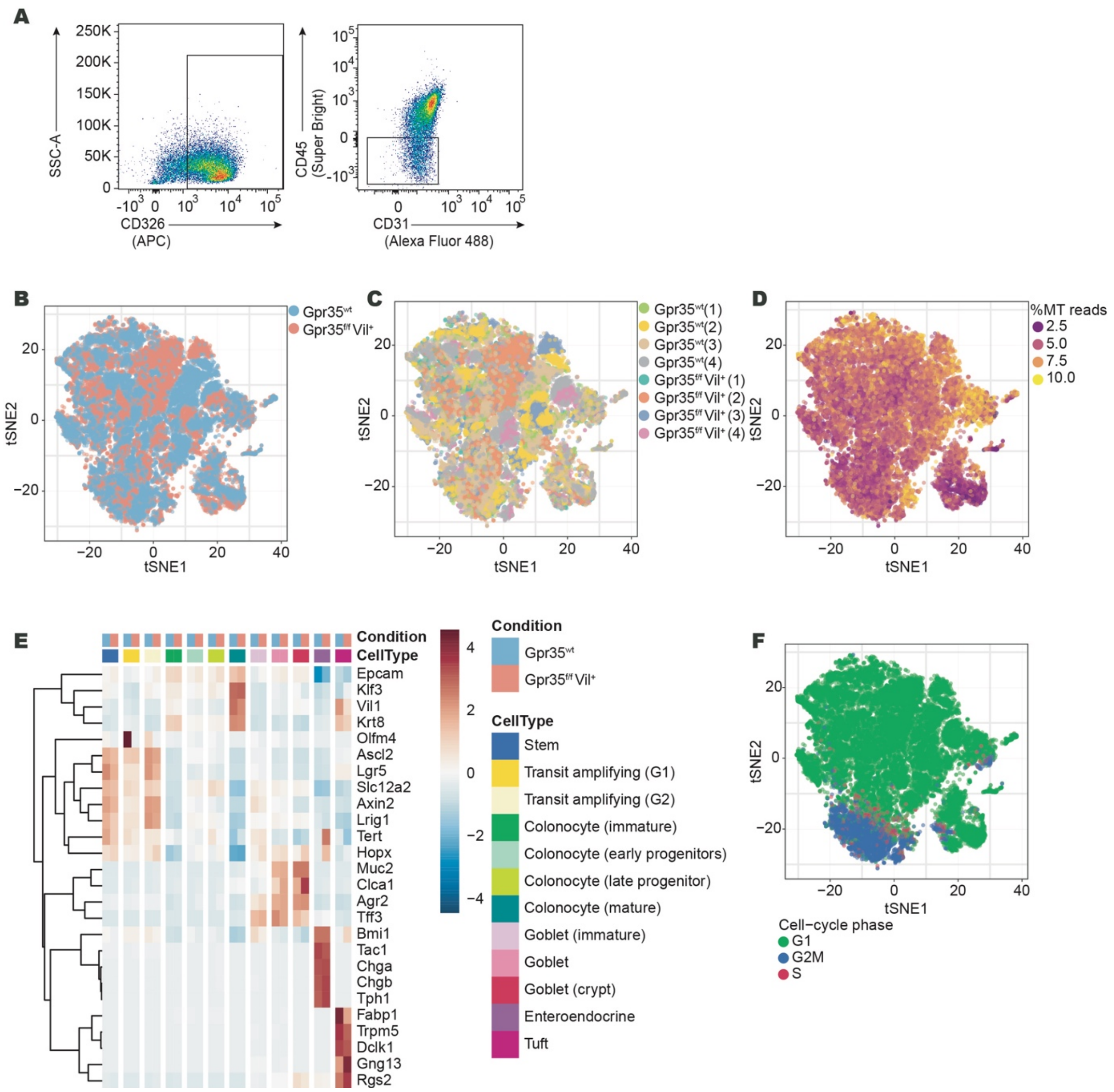
A
SSC-A
CD326 (APC)
CD45 (Super Bright)
CD31 (Alexa Fluor 488)
B
Gpr35wt
Gpr35f/f Vil+
tSNE1
tSNE2
C
Gpr35wt(1)
Gpr35wt(2)
Gpr35wt(3)
Gpr35wt(4)
Gpr35f/f Vil+ (1)
Gpr35f/f Vil+ (2)
Gpr35f/f Vil+ (3)
Gpr35f/f Vil+ (4)
D
%MT reads
2.5
5.0
7.5
10.0
E
Condition
CellType
Epcam
Klf3
Vil1
Krt8
Olfm4
Ascl2
Lgr5
Slc12a2
Axin2
Lrig1
Tert
Hopx
Muc2
Clca1
Agr2
Tff3
Bmi1
Tac1
Chga
Chgb
Tph1
Fabp1
Trpm5
Dclk1
Gng13
Rgs2
Stem
Transit amplifying (G1)
Transit amplifying (G2)
Colonocyte (immature)
Colonocyte (early progenitors)
Colonocyte (late progenitor)
Colonocyte (mature)
Goblet (immature)
Goblet
Goblet (crypt)
Enteroendocrine
Tuft
F
Cell−cycle phase
G1
G2M
S

Figure S3. Heterogeneity of colonic epithelial cells in *Gpr35$^{f/f}$Vil^{+}* mice

(A) Flow cytometry analysis of cells isolated from proximal colons of *Gpr35$^{f/f}$Vil^{+}* and *Gpr35^{wt}* co-housed littermates before scRNA-seq.

(B-D, F) *t*-SNE plot showing proximal colonic epithelial cells from *Gpr35$^{f/f}$Vil^{+}*(n = 4) and *Gpr35^{wt}* (n = 4) co-housed littermates assayed via scRNA-seq, and highlighting: (B) the distribution of *Gpr35$^{f/f}$Vil^{+}* and *Gpr35^{wt}* cells; (C) the distribution of cells from individual mice replicates; (D) the percentage of reads originating from mitochondrial genes; (F) the inferred cell-cycle phase.

(E) Heatmap showing the centered and scaled average expression across cell types and conditions of known cell type marker genes.

Figure S4

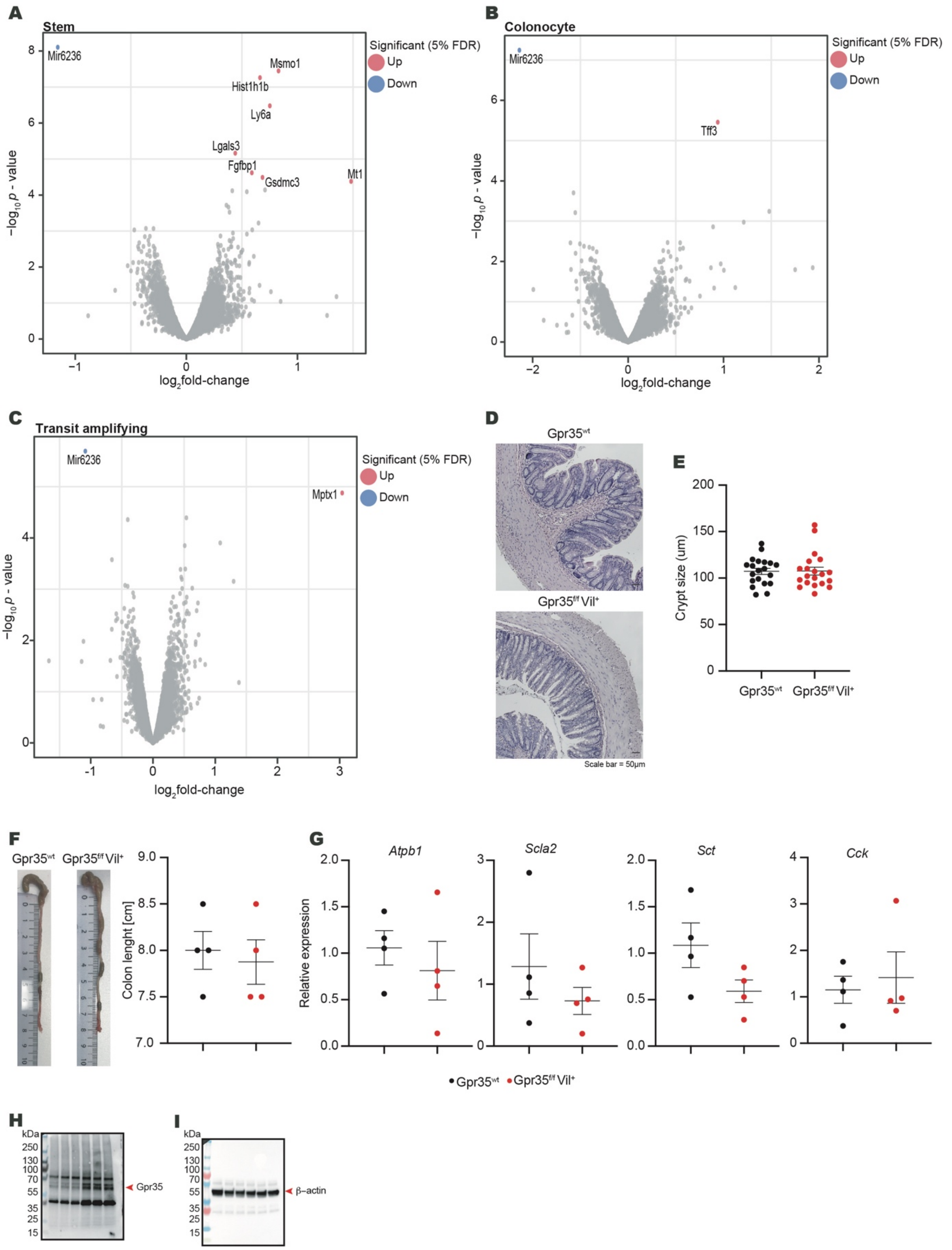
A
Stem
Significant (5% FDR)
Up
Down
Mir6236
Msmo1
Hist1h1b
Ly6a
Lgals3
Fgfbp1
Gsdmc3
Mt1
−log10 p - value
log2fold-change
B
Colonocyte
Significant (5% FDR)
Up
Down
Mir6236
Tff3
−log10 p - value
log2fold-change
C
Transit amplifying
Significant (5% FDR)
Up
Down
Mir6236
Mptx1
−log10 p - value
log2fold-change
D
Gpr35wt
Gpr35f/f Vil+
Scale bar = 50µm
E
Crypt size (um)
Gpr35wt
Gpr35f/f Vil+
F
Gpr35wt
Gpr35f/f Vil+
Colon lenght [cm]
G
Atpb1
Scla2
Sct
Cck
Relative expression
Gpr35wt
Gpr35f/f Vil+
H
kDa
Gpr35
I
kDa
β–actin

Figure S4. Increased pyroptotic signatures in goblet cells lacking epithelial *Gpr35*
(A-C) Volcano plot showing genes differentially expressed in *Gpr35*$^{f/f}$*Vil*$^{+}$ (n = 4) and *Gpr35*^{wt} (n = 4) (A) stem cells, (B) colonocytes or (C) transit amplifying cells. Figure legend similar to Figure 4A. Gpr35 was omitted from the plots for readability.
(D) Representative H&E images of proximal colon sections obtained from *Gpr35*$^{f/f}$*Vil*$^{+}$ and *Gpr35*^{wt} littermates.
(E) Crypt size quantification from (D).
(F) Colon lengths of 10-week-old *Gpr35*$^{f/f}$*Vil*$^{+}$ and *Gpr35*^{wt} mice.
(G) mRNA expression levels of *Atpb1, Scla2, Sct and Cck* measured by qRT-PCR in proximal colon samples obtained from *Gpr35*$^{f/f}$*Vil*$^{+}$ (n = 4) and *Gpr35*^{wt} littermates (n = 4).
Each dot represents one animal with medians. Data are represented as mean ± SEM *p ≤ 0.05, **p ≤ 0.01, ***p ≤ 0.001, ****p ≤ 0.0001 by Mann-Whitney in (E), (F) and (G).
(H-I) Whole membrane images of western blots represented in Figure 4F.

Figure S5

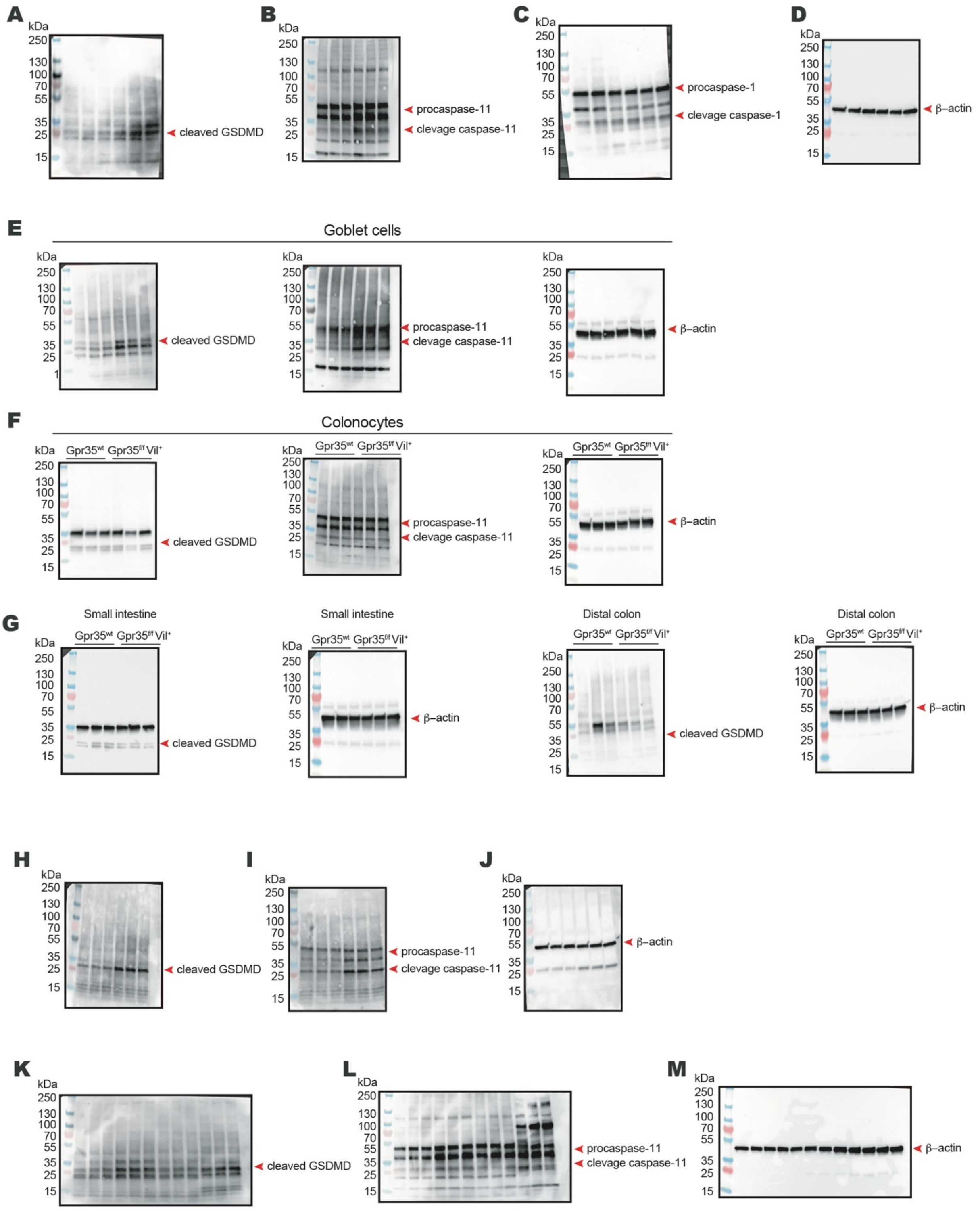
A
kDa
250
130
100
70
55
35
25
15
cleaved GSDMD
B
procaspase-11
clevage caspase-11
C
procaspase-1
clevage caspase-1
D
β–actin
E
Goblet cells
cleaved GSDMD
procaspase-11
clevage caspase-11
β–actin
F
Colonocytes
Gpr35wt
Gpr35f/f Vil+
cleaved GSDMD
procaspase-11
clevage caspase-11
β–actin
G
Small intestine
Distal colon
Gpr35wt
Gpr35f/f Vil+
cleaved GSDMD
β–actin
H
cleaved GSDMD
I
procaspase-11
clevage caspase-11
J
β–actin
K
cleaved GSDMD
L
procaspase-11
clevage caspase-11
M
β–actin

Figure S5. Pyroptosis upon *Gpr35* deletion is caspase-11 dependent
(A-M) Whole membrane images of western blots represented in Figure 5A-5I.

Figure S6

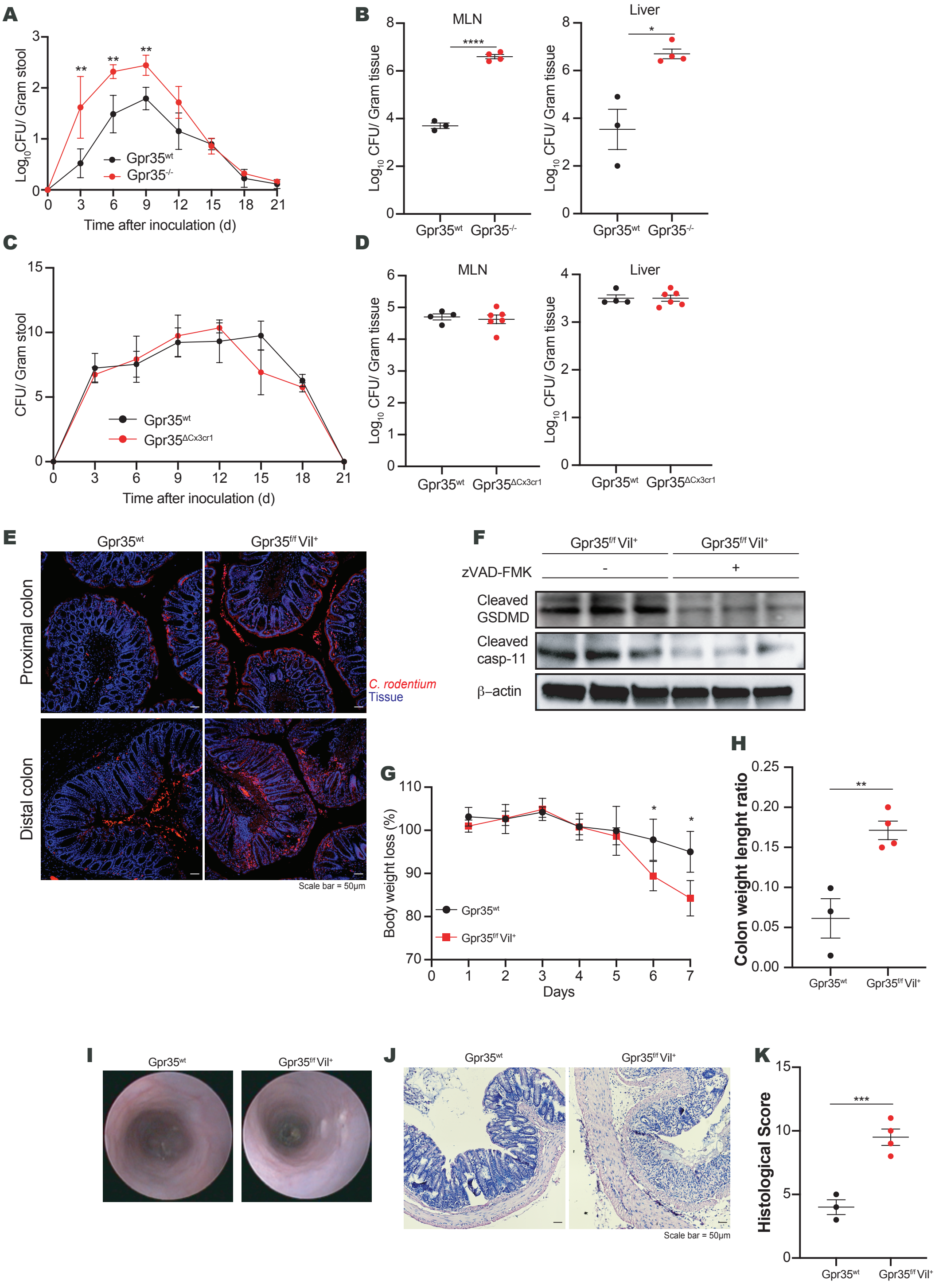

A
Log10CFU/ Gram stool
Time after inoculation (d)
Gpr35wt
Gpr35-/-
B
MLN
Liver
Log10 CFU/ Gram tissue
Gpr35wt Gpr35-/-
C
CFU/ Gram stool
Gpr35wt
Gpr35ΔCx3cr1
Time after inoculation (d)
D
MLN
Liver
Log10 CFU/ Gram tissue
Gpr35wt Gpr35ΔCx3cr1
E
Gpr35wt
Gpr35f/f Vil+
Proximal colon
Distal colon
C. rodentium
Tissue
Scale bar = 50μm
F
Gpr35f/f Vil+
Gpr35f/f Vil+
zVAD-FMK
-
+
Cleaved GSDMD
Cleaved casp-11
β-actin
G
Body weight loss (%)
Days
Gpr35wt
Gpr35f/f Vil+
H
Colon weight lenght ratio
Gpr35wt
Gpr35f/f Vil+
I
Gpr35wt
Gpr35f/f Vil+
J
Gpr35wt
Gpr35f/f Vil+
Scale bar = 50μm
K
Histological Score
Gpr35wt
Gpr35f/f Vil+

Figure S6. Epithelial GPR35 protects against *Citrobacter rodentium* infection
(A and B) *C. rodentium* CFU/g of (A) feces, (B) MLN and liver from *Gpr35*$^{-/-}$ (n = 4) and *Gpr35*^{wt} (n = 3) co-housed littermates.
(C and D) *C. rodentium* CFU/g of (C) feces, (D) MLN and liver from *Gpr35*$^{\Delta Cx3cr1}$ (n = 6) and *Gpr35*^{wt} (n = 4) co-housed littermates.
(E) Confocal microscopy images of proximal and distal colon sections infected obtained from *Gpr35*$^{f/f}$*Vil*$^{+}$ and *Gpr35*^{wt} co-housed littermates and infected with the *C. rodentium* mutant ICC 169 expressing the red fluorescent protein mRubby.
(F) Protein expression of cleaved GSDMD and cleaved caspase-11 in crypt samples isolated from the proximal colon obtained from *Gpr35*$^{f/f}$*Vil*$^{+}$ mice treated with zVAD-FMK (n = 3) or not (n = 3).
(G) Body weight changes of *Gpr35*$^{f/f}$*Vil*$^{+}$ (n = 4) and *Gpr35*^{wt} (n = 3) co-housed littermates (normalized to initial weight).
(H) The colon weight/length ratio is represented as milligrams per centimeter of colon.
(I) Endoscopic images of *Gpr35*$^{f/f}$*Vil*$^{+}$ and *Gpr35*^{wt} mice.
(J) Representative H&E images of proximal colon sections obtained from *Gpr35*$^{f/f}$*Vil*$^{+}$ and *Gpr35*^{wt} co-housed littermates.
(K) Histology scores quantified from (I).
Each dot represents one animal with medians. Data are represented as mean ± SEM $^{*}p \leq 0.05$, $^{**}p \leq 0.01$, $^{***}p \leq 0.001$, $^{****}p \leq 0.0001$ by two-way ANOVA with Tukey's multiple comparisons test in (A), (C) and (F) or Mann-Whitney in (B), (D), (H) and (K).

Table S2: q-PCR primers.

Muc2 F	CCC AGA AGG GAC TGT GTA TG
Muc2 R	TGC AGA CAC ACT GCT CAC A
Atpb1 F	ACC TAC ACT TAA TCT CTA TGC
Atpb1 R	GAA CAC TGC TCA CTA CAA
Slc2a1 F	TAT CTC CAC ACT GTA GTC
Slc2a1 R	CAG AGT TCG GTA TTA GTG
Cck F	AGG AAA CAA CCA CAC ATA CG
Cck R	AGC ATA GCA ACA TTA GGT CTG
Sct F	CTG CTG TTG CTG CTG CTG
Sct R	CAT TCC GTC TGA GTG TCT TGG
Gapdh F	CAT CAA GAA GGT GGT GAA GC
Gapdh R	CCT GTT GCT GTA GCC GTA TT

Table S3: 16S primers.

Forward	GTG**N**CAGCMGCCGCGGTAA
Reverse	GGACTACHVGGGT**N**TCTAA
